# The Biomarker Profile of Alzheimer’s Disease for Disease-Modifying Treatment Eligibility: Questions and Debates

**DOI:** 10.3390/ijms26199531

**Published:** 2025-09-29

**Authors:** Athanasia Athanasaki, Ioanna Tsantzali, Aikaterini Theodorou, Amalia Michalopoulou, Vasilios C. Constantinides, Fotini Boufidou, John S. Tzartos, Panagiota-Eleni Tsalouchidou, Christina Zompola, Sotirios G. Paraskevas, Anastasios Bonakis, Sotirios Giannopoulos, Georgios Tsivgoulis, Elisabeth Kapaki, George P. Paraskevas

**Affiliations:** 1 2nd Department of Neurology, School of Medicine, National and Kapodistrian University of Athens, “Attikon” General University Hospital, 12462 Athens, Greece; athanasia.athan@yahoo.gr (A.A.); docjo1989@gmail.com (I.T.); katetheo24@gmail.com (A.T.); aliam2204@gmail.com (A.M.); jtzartos@gmail.com (J.S.T.); pania.tsalouchidou@gmail.com (P.-E.T.); chriszompola@yahoo.gr (C.Z.); sotirispar5@gmail.com (S.G.P.); bonakistasos@med.uoa.gr (A.B.); sgiannop@uoi.gr (S.G.); tsivgoulisgiorg@yahoo.gr (G.T.); 2 1st Department of Neurology, School of Medicine, National and Kapodistrian University of Athens, Neurochemistry and Biological Markers Unit, “Eginition” Hospital, 11528 Athens, Greece; vassilis.kon@hotmail.com (V.C.C.); fboufidou@med.uoa.gr (F.B.); ekapaki@med.uoa.gr (E.K.)

**Keywords:** Alzheimer’s disease, biomarkers, amyloid beta, tau protein, phospho-tau protein, lecanemab, donanemab, disease modifying treatments

## Abstract

Alzheimer’s disease (AD) is the most common cause of cognitive decline; currently, anti-amyloid monoclonal antibodies are available for clinical use as disease-modifying treatments, while many other substances are being tested in clinical trials. Molecular biomarkers for AD have been studied for more than two decades, and various guidelines and diagnostic recommendations have been published. However, there are still questions and controversies about the biomarker profile needed to confirm AD and the eligibility for such established treatments and clinical trials. Is amyloid positivity sufficient for eligibility, or is a biomarker for tau biochemistry/pathology also needed? What is the role of hybrid ratios combining amyloid and tau? Should we rely on plasma biomarkers alone? This review aimed to describe and discuss such questions and controversies.

## 1. Introduction

Alzheimer’s disease (AD) is the most common neurodegenerative disease and cause of cognitive decline [1]. It accounts for ~60–70% of cases; by 2030, patients with AD are expected to reach 75 million worldwide, posing a major health and socioeconomic problem [2]. The main biochemical and pathological characteristics of AD include the extracellular polymerization of amyloid beta peptide with 42 amino acids (Aβ_42_) forming amyloid plaques [3] and the intracellular polymerization of hyperphosphorylated tau protein in the form of paired helical filaments evolving to neurofibrillary tangles [4]. The interplay between the two converging mechanisms [5] leads to AD [6].

Until a few years ago, AD therapeutics were based on symptomatic treatments introduced ~25 years ago [7]. Since the amyloid pathway is an early and key pathogenic mechanism, as well as a potential therapeutic target [8], anti-amyloid monoclonal antibodies have been recently introduced in AD therapeutics as the first disease-modifying treatments [7,8,9,10]. Currently, Lecanemab [11] and Donanemab [12] are the two anti-amyloid antibodies fully approved in the USA by the Food and Drug Administration and in Europe by the European Medicines Agency. They are approved for use in early AD, i.e., mild cognitive impairment (MCI) or mild dementia due to AD; appropriate use recommendations thought to be useful in clinical practice [13] have been published from authorities in various states, including the U.S.A. [14,15], France [16], Canada [17], and South Korea [18].

But how is AD diagnosed during life? It has been observed that clinical diagnosis may be accompanied by a high clinicopathological concordance (>90%) when typical cases without comorbidities, fulfilling even the older probable criteria of AD [19], are examined by specialists in tertiary/academic centers [20]. However, this is not always true; in early or atypical cases, in the presence of comorbidities, and in the community (approach by non-experts), diagnostic accuracy may decrease to <75% [21]. In general, it has been estimated that ~25–30% of patients clinically diagnosed with AD will suffer from a non-AD disorder at post-mortem pathological examination [22]. On the other hand, post-mortem examination will reveal the presence of AD (co)pathology in ~39% of cases of patients with a clinical diagnosis of non-AD disorder [23]. Thus, it is not surprising that the chance of in vivo misdiagnosis was ~1/3 in a more recent pathologically confirmed study from an academic center [24]. The newer disease-modifying anti-amyloid antibodies are (a) effective in cases with early AD, i.e., exactly in the population where clinical diagnostic accuracy may be relatively lower, and (b) not free of safety issues, including amyloid-related imaging abnormality (ARIA) [11,12,25]. Thus, the maximal possible diagnostic accuracy should be ensured before considering their administration. Over the last 25 years, classical cerebrospinal fluid (CSF) [26,27] and molecular biomarkers visualized via positron emission tomography (PET) [28] have become the gold standard in vivo diagnosis of AD. They have been incorporated into diagnostic criteria and guidelines [29,30,31,32,33,34], resulting in pathologically verified improvement of in vivo diagnostic accuracy [24]. Research on biomarker-based diagnosis has intensified in the context of anti-amyloid treatment approval [35,36,37].

There are many extensive, comprehensive, and very informative reviews and recommendations on the use of various biomarkers for AD, many of which are cited in this study. A search gave a total of 886, 626, and 1602 results for CSF [38], PET [39], and blood-based biomarkers [40], respectively. However, despite improved diagnostic abilities [37], there are still controversial issues and debates among various authorities that publish diagnostic and classification criteria for AD [41,42,43,44,45]. The aim of this work was not to present just another review but to present some critical points and questions related to the molecular biomarker-based approach in diagnosing AD and identifying patients eligible for approved or investigational disease-modifying treatments.

## 2. Classical Biomarkers with Molecular Specificity for AD

Classical biomarkers with molecular specificity for AD are related to the two fundamental biochemical and pathological hallmarks of AD: Aβ formation and deposition in amyloid plaques and tau hyperphosphorylation and the formation of paired helical filaments and neurofibrillary tangles.

### 2.1. CSF Markers

Aβ_42_ levels are decreased in the CSF of patients with AD, inversely related to amyloid plaque burden [46]. Amyloid beta peptide with 40 amino acids (Aβ_40_) can also be determined, and the Aβ_42_/Aβ_40_ ratio may be a better indicator of amyloid plaque formation [47], being preferable to Aβ_42_ alone [48]. Thus, Aβ_42_/Aβ_40_ shows molecular specificity for the amyloid component of AD.

Increased levels of hyperphosphorylated tau protein are considered a marker of the tauopathic component of AD [49]. Tau hyperphosphorylated at a threonine residue at position 181 (τ_P-181_) is the most widely used form; however, other hyperphosphorylated tau forms can be quantified in the CSF, including τ_P-217_ and τ_P-231_ [50], with τ_P-217_ having some possible advantages over τ_P-181_ [51]. All of the above hyperphosphorylated tau forms become abnormal early in the disease process, when tau molecules and small aggregates are still soluble (before neurofibrillary tangle formation), as a result of the amyloid cascade, due to amyloid-triggered activation of kinases such as cyclin-dependent kinase-5 (CDK-5), glycogen synthase kinase-3β (GSK-3β), and mitogen-activated protein kinases (MAPKs) [52,53]. Thus, these forms may be viewed as markers of the amyloid-triggered initiation of the tauopathic process and as “indirect markers” of amyloidogenesis. In this context, CSF τ_P-217_ (especially the ratio of the phosphorylated to total form) may be a slightly better indicator of amylogenesis than the Aβ_42_/Aβ_40_ ratio [54]. Recently, CSF τ_P-212_, which may be related to the action of dual-specificity tyrosine phosphorylation-regulated kinase 1A, was found to be increased early in the course of AD, either sporadic or due to Down syndrome [55].

As tauopathy progresses, tau aggregates become insoluble, and tangle pathology emerges. At this time, other tau forms increase in the CSF, including the tau microtubule-binding region containing residue 243 (MDBR243) [56] and τ_P-205_ [57], which enables AD staging [58].

### 2.2. PET Markers

PET for Aβ with various tracers, such as florbetaben, florbetapir, and flutemetamol, provides imaging evidence of amyloid deposition [59]. It can be interpreted visually or by Standardized Uptake Value Ratios (SUVRs) or centiloids for detecting neuritic plaque load corresponding to Thal phases 3–5 [60]. Thus, PET for Aβ generally correlates with the Thal phases; however, it cannot detect the very early phases of amyloidogenesis and early Thal phases, and a centiloid level of at least 28–30 should be reached for diagnostic purposes [61].

On the other hand, PET for tau deposition (neurofibrillary tangle formation) generally follows Braak stages, usually requiring a Braak stage of at least III [62], whilst for diagnostic purposes, ^18^F-flortaucipir PET detects stages ≥ IV [63]. The severity of tau PET abnormality correlates positively with the AD progression rate [64]. Furthermore, while in PET for Aβ, the amyloid distribution pattern is usually similar in all AD patients, the tau load distribution in tau PET is not the same and may be either typical (as expected by Braak staging) or atypical, showing a posterior, left predominant, or hippocampal sparing pattern [65]. Thus, PET for tau deposition may correlate with both the clinical picture (typical amnestic or atypical presentations) and AD severity.

## 3. Other Biomarkers Typically Used

### 3.1. Markers of Neurodegeneration

Cerebrospinal fluid τ_T_ [66,67] and NfL [68] are long-known markers of neuronal/axonal degeneration, and their levels are usually increased in AD [4]. However, they are not specific for AD and may be increased in other neurodegenerative disorders [69].

Positron emission tomography (PET) with 18F-fluoro-deoxy-glucose (FDG) can reveal hypometabolic brain areas. In AD, hypometabolism occurs typically in the parietotemporal association cortices, posterior cingulate cortex, and precuneus [70], and this helps in the differential diagnosis from other neurodegenerative disorders [33]. However, deviations from the typical topography occur in atypical clinical presentations of AD, posing difficulties in the diagnostic process, for example, between frontotemporal dementias and the frontal variant of AD [70,71]. Single-Photon Emission Computed Tomography (SPECT) with ^99m^Tc-Hexamethylpropylen-amine Oxime (HMPAO) for assessing hypoperfusion may be a useful alternative, depending on availability issues [72]. Recently, 3-Tesla Magnetic Resonance Imaging (3T MRI) with arterial spin labeling (ASL) quantifying cerebral blood flow has emerged as another alternative [73], more affordable, and radiation-free imaging method, suitable for longitudinal follow-up and with a diagnostic accuracy that may be comparable to that of PET [74].

Brain atrophy observed in MRI or even CT is another useful marker of neurodegeneration, and the topography may be helpful in the (differential) diagnosis of AD. Volumetry may have some advantages [75], but visual scales assessing the atrophy of hippocampal formation, such as the long-known Medial Temporal Atrophy (MTA) scale [76] or, more recently, the Entorhinal Cortex Atrophy (ERICA) scale [77,78], are easy-to-use tools for everyday practice.

It is worth noting that the term neurodegeneration encompasses many components, including neuronal and/or axonal and/or neuropil dysfunction/loss, hypometabolism and hypoperfusion, and central and cortical atrophy [41]. Therefore, the above markers are not equivalent and may show different patterns of evolution during the disease process, with atrophy usually being the last to emerge [79].

### 3.2. Markers of Other Primary Co-Pathologies

For vascular co-pathology, MRI or CT are the usual imaging methods. In case of large vessels and/or lacunar infarcts, the vascular nature of the lesions is easily recognized. However, white matter hyperintensities should not always be attributed to cerebral small vessel disease in AD. It has been observed that anterior (frontal) white matter lesions may be associated with both vascular pathology and AD neurodegenerative changes (tau-related axonal loss and Aβ burden) [80,81]; thus, they may be related to mixed cognitive impairment (AD + vascular). On the contrary, posterior (parietal) periventricular lesions are related to cortical AD pathology [82] and, if associated with hippocampal and temporal atrophy, may even serve as early biomarkers of AD [81]. A juxtacortical multisport pattern of white matter MRI hyperintensities and lobar microbleeds points to cerebral amyloid angiopathy [81,83].

Various methods of assessing CSF α-synuclein (α-syn) have been tested [84,85,86], but the seeding amplification assay is the most promising [87,88,89]. PET for α-syn has also been studied [28]. Similarly, CSF and PET assessments of transactive-response DNA-binding protein-43 (TDP-43) have been studied [90,91], but it is unknown whether they can be used effectively to identify TDP-43 co-pathology in patients with AD [92]. TDP-43 accumulation is the hallmark of limbic predominant age-related TDP-43 encephalopathy (LATE), an entity characterized by AD-like symptoms mainly in patients over 80 years old, with distinct neuropathological stages initiated by the amygdala [91]. Co-pathology identification is significant since TDP-43 inclusions can occur up to 50% in AD and may be closely related to the disease’s pathogenesis and prognosis [91,92].

## 4. Plasma Biomarkers

The above-described biomarkers are either CSF-based (requiring lumbar puncture, a generally safe but minimally to moderately invasive procedure) or imaging-based (mainly PET). Blood-based (plasma) biomarkers have received significant attention recently due to their noninvasiveness and reduced costs compared to CSF and PET biomarkers [93,94].

Plasma Aβ_42_/Aβ_40_ ratio [95,96], plasma τ_P-181_ [97,98], plasma τ_P-231_ [99], plasma τ_P-212_ [55], and especially plasma τ_P-217_ [95,96,99,100,101,102] are early markers of AD; as in CSF, the above phosphorylated tau forms are good indicators of not only amyloid-triggered tau hyperphosphorylation but also amyloidogenesis [95,96,99], with a sensitivity comparable to that of CSF Aβ markers and better than CSF τ_P-181_ [103]. It has been suggested that plasma levels of the endogenous cleaved tau microtubule-binding region containing residue 243 (eMBDR-tau243) increase when tangle pathology appears [104].

Plasma levels of NfL serve as a nonspecific marker of neurodegeneration in AD [105]. Therefore, NfL assessment could serve as a valuable prognostic marker in AD, considering its association with brain atrophy, especially if it were feasible to acquire repetitive values throughout the course of the disease [105].

Neuroinflammation markers, such as Glial Fibrillary Acidic Protein (GFAP), may be assessed in plasma [106], and it has been observed that plasma GFAP levels may perform diagnostically better than CSF levels [107]. Hence, the response of plasma GFAP levels to monoclonal antibodies has been studied in crucial randomized trials [11,12], and there is evidence that their increase, as a result of reactive astrocytosis, could accompany much earlier stages of AD pathology. More specifically, findings that contain uncertainty suggest a relation to the amyloid cascade, even in the preclinical phase of the disease [106,107].

## 5. Alternative Biomarkers

Some biomarkers have been introduced into research relatively recently or have been long known to have special advantages in studying various aspects of AD pathology [26]. They could have the potential to be helpful either clinically or to study (in clinical trials) the possible benefits of new treatments in aspects other than Aβ or tau proteinopathy.

### 5.1. Biomarkers of Amyloidopathy or Tauopathy

Soluble Aβ oligomers, formed very early in the amyloid cascade process, are thought to be neurotoxic through their ligand-like action [108]. They can be quantified in CSF [109] and plasma [110] and have the potential to offer very early diagnosis [111]. Recently, a portable apparatus detecting Aβ oligomers has been tested, promising easy diagnosis with low costs, even at the site of primary care [112]. In a similar context, CSF quantification of tau and α-syn oligomers is also being tested as a diagnostic tool, possibly useful in the differential diagnosis of dementia-related and Parkinsonian neurodegenerative disorders [113].

In addition to the only approved tracer for PET, ^18^F-flortaucipir, other tracers are being tested [114], and there is evidence that some may outperform flortaucipir, showing increased selectivity for affected brain areas [115].

### 5.2. Biomarkers of Microglia Activation and Other Parameters of Neuroinflammation

Microglia are activated early in Alzheimer’s disease in an attempt to remove aggregating Aβ but with ongoing increases in Aβ burden, impairment in microglial phagocytic activity, and chronic dysregulated neuroinflammation occur, leading to neurodegeneration [116]. However, this phenomenon is nonspecific and may occur in many other neurodegenerative conditions. Triggering Receptor Expressed on Myeloid cells 2 (TREM2) is a marker of microglia activation, which has received much attention recently due to its involvement in AD mechanisms, its potential as a diagnostic marker, and its role as a possible therapeutic target [106]. The CSF levels of soluble TREM2 (sTREM2) are increased in early AD [117,118] and could be related to discrepancies between Aβ_42_ levels and the τ_P-217_/Aβ_42_ ratio [119]. sTREM2 can also be measured in plasma [120] and may be associated with concomitant cerebrovascular disease [121]. However, abnormal levels of sTREM2 may be observed in other neurodegenerative disorders [122].

CSF or plasma levels of chitinase 3-like protein 1 (YKL-40) [123,124,125] and numerous other markers, including Interleukin-1β (IL-1β), Tumor Necrosis Factor-alpha (TNF-α), and S100, involved in the interplay between microglia, astrocytes, and inflammation, are being extensively studied both as diagnostic biomarkers and possible targets of individualized therapies [126,127,128].

Recently, CSF levels of soluble α-Klotho (sαKl), a protein involved in antioxidant and neuroprotective mechanisms, have been reported to be lower in dementia due to AD compared to controls [129].

### 5.3. Biomarkers of Synaptic Loss

Synaptic dysfunction and loss are integral parts of Alzheimer’s neuropathology [130]. Among other molecules, Synaptotagmin (SYT-1) [131], Synaptosomal-Associated Protein 25 (SNAP-25) [132,133], Postsynaptic Density Protein 95 (PSD-95) [133], Neurogranin [133,134], and Vesicle-Associated Membrane Protein 2 (VAMP-2) [135] have been tested as possible markers of synaptic loss in AD.

Brain-Derived Neurotrophic Factor (BDNF) is a neurotrophin supporting neuronal and synaptic integrity and plasticity. Low levels have been reported in Alzheimer’s disease, associated with Aβ- and tau-related mechanisms, neuroinflammation, and apoptosis, but BDNF may be involved in many other neurological and psychiatric disorders [136].

It has been suggested that combining various classical biomarkers with markers of neuroinflammation and synaptic loss may prove diagnostically helpful and even outperform classical biomarkers alone [128]. However, in an umbrella review, only blood-based YKL-40 yielded convincing results, while many others were suggestive than established proof [137].

## 6. Genetic Biomarkers

Apolipoprotein E (*APOE*) genotyping is not uncommonly performed in cognitively affected patients. It is a susceptibility gene for AD, present in three allelic forms: ε2, ε3, and ε4 [138]. The presence of at least one ε4 allele is a major risk factor for AD [138], while the ε2 allele may be protective [139]. Recently, it has been suggested that although ε4 heterozygosity is a risk factor, ε4 homozygosity may represent a distinct genetic type of AD [140]. This notion has been challenged [44] since it has been observed that the concomitant presence of a loss-of-function mutation in the Sortilin-Related Receptor 1 gene (*SORL1*) is necessary for this to occur [141]. Thus, in addition to evaluating the risk of AD, *APOE* and/or *SORL1* testing could be diagnostically helpful.

Nevertheless, *APOE* genotyping before initiating anti-amyloid treatment is considered significant for AD patients since the presence of ε4 increases the risk of side effects (amyloid-related imaging abnormalities), and in the case of ε4 homozygosity, these drugs may be contraindicated [14,15,16]. It is also significant in clinical trials for newer drugs targeting ε4 [142].

Mutations in the gene encoding TREM2 may be related to neurodegeneration and increase the risk of AD [143].

Down syndrome is a specific genetic type of AD [144] for which anti-Aβ antibody treatment is contraindicated due to the increased presence of cerebral amyloid angiopathy [14]. These treatments are also contraindicated in Amyloid Precursor Protein gene (*APP*) mutations associated with cerebral amyloid angiopathy [14].

## 7. Profiles and Ratios

The National Institute of Aging—Alzheimer’s Association (NIA-AA) research framework (2018) introduced the so-called AT(N) system for the diagnosis and classification of AD [41]. A stands for amyloid biomarkers, and A^+^ and A^−^ indicate positive (abnormal) and negative (normal) results of the biomarker used for amyloids, respectively. T stands for tauopathy biomarkers (tau phosphorylation, tangle formation), with T^+^ and T^−^ indicating abnormal and normal results of the biomarker tested, respectively. Finally, (N) stands for neurodegeneration (neuronal or axonal dysfunction or loss), with (N) and (N) indicating abnormal and normal results of the corresponding tests, respectively.

Biomarkers widely used (or suggested for use) currently in AD diagnosis and staging are summarized in Table 1, according to the 2018 NIA-AA research framework and the 2024 Alzheimer’s Association (AA) revised classification system [41,43]. Since biomarkers for neurodegeneration or neuroinflammation are nonspecific, and vascular biomarkers or α-syn indicate possible co-pathologies, only those indicating amyloid- and tau-related biochemistry/pathology are currently considered suitable for AD diagnosis and staging [27].

**Figure 1:**
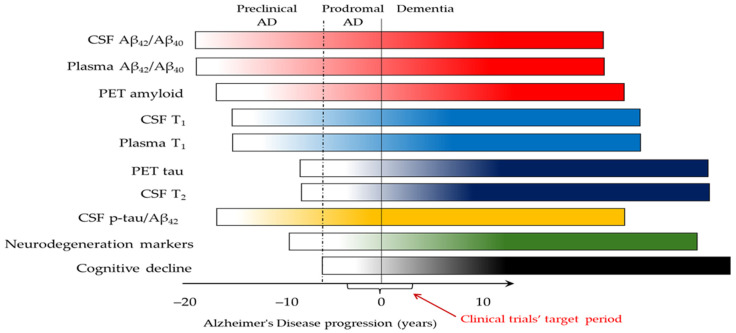
Theoretical diagram of biomarker changes during Alzheimer’s disease evolution and progression [41,43,103,144,145,146,147,148]. The changes in biomarkers follow the smooth increase in color intensity throughout the course of AD. The red arrow shows the period that is the target of monoclonal antibodies’ clinical trials. Deviations may occur in some patients based on genetic or other factors, as well as the biomarker tests used. Cerebrospinal fluid (CSF) T_1_ refers to τ_P-181_, τ_P-217_, and τ_P-231_. Plasma T_1_ refers to τ_P-217_, τ_P-181_, and τ_P-212_. CSF T_2_ mainly refers to τ_P-205_ and MDBR-τ_243_. For simplicity reasons, neurodegeneration markers are shown together in one bar, although they are not equivalent and may become abnormal at relatively different time points.

Cumulative data from many studies [103,144,145,146,147,148] lead to the general notion that CSF or plasma Aβ_42_/Aβ_40_ are the first to become abnormal very early in the disease process and earlier than PET for Aβ up to ~19 years before symptom onset (Figure 1). The levels of CSF τ_P-181_, τ_P-217_, τ_P-231_, and τ_P-212_ may start to increase around the first indication of abnormality in Aβ PET, and they are usually already abnormal when Aβ PET reaches the diagnostic threshold (28–30 centiloids). Thus, the above phospho-tau (“T”) forms [41] are now termed “T_1_”, and, together with Aβ (A) markers, they are classified as “core 1” biomarkers [43]. With disease progression, insoluble forms of tau accumulate in the form of neurofibrillary tangles, at which point CSF or plasma τ_P-205_ and MBDR-τ_243_ become abnormal, and tangle pathology becomes evident in PET for tau. These “T” biomarkers are now classified as “T_2_” and “core 2” biomarkers [43].

According to the 2018 NIA-AA criteria, AD can be diagnosed when the biomarker profile is A^+^T^+^, while the A^+^T^−^ profile is classified as Alzheimer’s continuum since only amyloid biomarkers may be positive very early in the disease process, with T biomarkers remaining negative [41]. According to the 2024 AA revised criteria, an abnormal core 1 biomarker either A (PET for Aβ, or CSF Aβ_42_/Aβ_40_) or plasma T_1_ (τ_P-217_, good indicator of Aβ pathology) is sufficient for AD diagnosis since the vast majority of A^+^ patients (~90% with positive PET for Aβ) will prove to have AD at autopsy [43]. Indeed, this is true, but it is not always the case.

Firstly, the A^+^ profile is heterogeneous, comprising different subgroups of patients with typical amnestic AD, AD mixed with other pathologies, or vascular cognitive decline [149]. The A^+^T^−^ profile is also heterogeneous. Many such patients may suffer from early presymptomatic or even mildly symptomatic AD in which the T biomarkers have not become abnormal yet [145]. Additionally, AD with atypical clinical presentations [150,151,152] or mixed AD [153] may present with marginal or normal CSF τ_P-181_ levels. The A^+^T^−^ profile may also be observed in other non-AD disorders such as dementia with Lewy bodies (DLB) [154], subcortical small vessel disease, various tauopathies [155,156], Creutzfeldt–Jakob disease [157], limbic-predominant age-related TDP-43 encephalopathy [158], subcortical small vessel disease [159], and cerebral autosomal dominant arteriopathy with subcortical infarcts and leukoencephalopathy (CADASIL) [160]. Ιn such clinical settings, with equivocal CSF profiles, the Aβ_42_/Aβ_40_ ratio, instead of Aβ_42_ alone, led to a reliable diagnosis in ~50% of cases [161], but not in all, and diagnostic uncertainty may remain. A proportion of A^+^T^−^ patients may remain A^+^T^−^ for at least 5 years, showing some clinical and genetic differences compared to those with A^+^T^+^ [162]. Recently, an amyloid predominant subtype of Alzheimer’s neuropathological change (~10%) has been described, with patients progressing to amyloid Thal phase 5 while remaining in tau Braak stages I–II and not progressing up to stage VI, as would be expected for AD; these patients may present with clinical and genetic differences compared to those with typical AD pathology [163]. Furthermore, some patients with amyloid pathology may never progress clinically [164]. Taken together, the above findings indicate that a percentage of A^+^ or A^+^T^−^ patients, despite showing amyloid pathology, may deviate from what is expected in AD.

To increase the diagnostic performance of biomarkers, hybrid ratios of different biomarkers have been used, such as CSF τ_P-181_/Aβ_42_, CSF τ_T_/Aβ_42_, and plasma τ_P-217_/Aβ_42_ [165,166,167,168,169,170]. Since the nominator increases and the denominator decreases, the ratio change is augmented, resulting in good discrimination between AD and non-AD disorders. The CSF τ_P-181_/Aβ_42_ and τ_T_/Aβ_42_ ratios may be helpful in cases with marginal or conflicting results from individual biomarkers [166] and in discriminating between likely AD and likely non-AD patients within the A^+^T^−^ profile [171]. However, these ratios could sometimes become abnormal due to a significant reduction in only Aβ_42_, i.e., in the absence of any evidence of tau-related biochemistry. These CSF hybrid ratios and the CSF Aβ_42_/Aβ_40_ ratio, also included in Table 1, are classified independently as “core 1” biomarkers according to the 2024 AA revised criteria, reflecting a well-established AD neuropathological change, even in asymptomatic individuals [43]. The importance of the CSF Aβ_42_/Aβ_40_ ratio, which identifies amyloid pathology with adequate accuracy, has already been mentioned as a key initial change over the course of AD in relation to classic fluid and imaging biomarkers [147,148]. Ultimately, plasma τ_P-217_/Aβ_42_, which has recently received FDA approval, established encouraging data in the cognitive impaired population using the two-cut-off approach, but it needs further investigation to strengthen its clinical utility [170].

In the approval study of Lecanemab (Clarity AD), evidence of amyloid positivity was required for recruitment (PET or CSF Aβ_42_) [11], while for Donanemab (TRAILBLAZER-ALZ 2), PET scans for both Aβ and tau were required [12]. According to appropriate use recommendations for Lecanemab, an abnormal PET for Aβ or CSF findings of increased p-tau and reduced Aβ_42_ (increased p-tau/Aβ_42_) is required [14], while for Donanemab, amyloid positivity by an abnormal PET for Aβ or by reduced CSF Aβ_42_/Aβ_40_ or, alternatively, by abnormal CSF τ_P-181_/Aβ_42_ and τ_T_/Aβ_42_ ratios are required [15]. Canadian [17] and Korean [18] guidelines are similar to the appropriate use recommendations for Lecanemab and Donanemab. However, the French appropriate use recommendations for Lecanemab show some deviations since they recommend the CSF A^+^T^+^ profile, and in cases with inconclusive CSF results or, alternatively, an abnormal PET for Aβ [16]. In fact, similar recommendations are advocated by the International Working Group (IWG) criteria for the diagnosis of AD [42,44].

Thus, there are still debates over the definition and biomarker profile of AD, i.e., amongst the 2018 NIA-AA criteria [41], 2024 AA revised criteria [43], and IWG criteria [42,44]. However, this may be very important since it has been observed that different criteria may result in discordant diagnoses in ~42% of patients, especially when only one biomarker is abnormal [172].

## 8. Discussion

Currently, the most widely used biomarkers in everyday practice for AD diagnosis are the classical or core biomarkers (CSF or PET). Depending on availability, familiarity, acceptance, contraindications, cost, and reimbursement issues, either CSF or PET may be preferred in various countries for confirming AD and eligibility for anti-amyloid treatments or enrolment in experimental trials. However, after roughly 25 years of research on classical biomarkers, there are still controversies.

- Should we use the AT(N) classification system according to the 2018 NIA-AA guidelines [41] or the 2024 AA revised criteria [43]?
- Should we use the AA revised guidelines [43] or the IWG recommendations [44]?
- Should the profile be both A^+^ and T^+^ (either T_1_^+^ or T_2_^+^), or is A^+^ sufficient?
- Should we rely on the τ_P-181_/Aβ_42_ and τ_T_/Aβ_42_ ratios and when?

**Figure 2:**
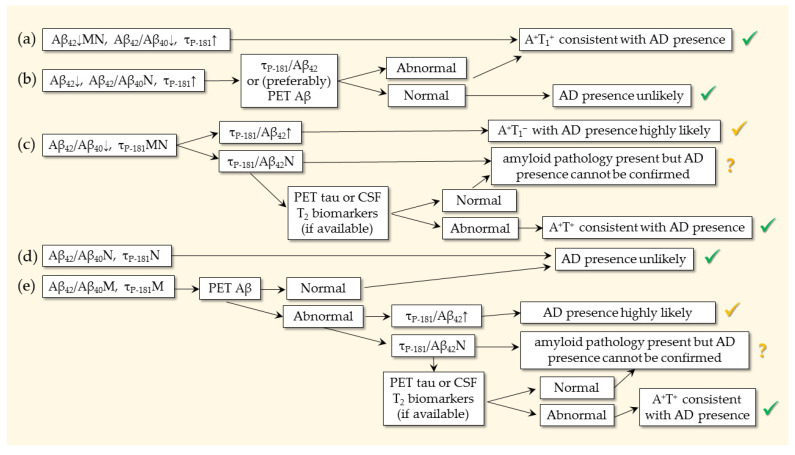
Use of biomarkers for the confirmation of AD presence in 5 scenarios, starting from CSF analysis. N: normal; M: marginal. Green “O.K. symbols” indicate confirmation. Yellow “O.K. symbols” indicate the likely presence of AD. Question marks indicate the presence of amyloid pathology, but AD (additional occurrence of tau pathology) cannot be verified. In scenario (**a**), the typical CSF profile of AD is present (A^+^T^+^ or A^+^T_1_^+^). In scenario (**b**), Aβ_42_/Aβ_40_ is normal, raising questions about the presence of amyloid pathology, despite the abnormality of both Aβ_42_ and τ_P-181_. In this case, a PET scan for Aβ or (if not available) the τ_P-181_/Aβ_42_ ratio is required to reach a conclusion. In scenario (**c**), the A^+^T^−^ profile is present with abnormality only in the Aβ_42_/Aβ_40_ ratio (or alternatively the amyloid PET). In this case, the τ_P-181_/Aβ_42_ ratio can be used, but if normal, a T_2_ biomarker (if available) may be required. In scenario (**d**), the A^−^T^−^ (A^−^T_1_^−^) profile is inconsistent with AD. In scenario (**e**), both molecular biomarkers are marginal. In such patients, the τ_P-181_/Aβ_42_ ratio and/or PET scans for Aβ and/or tau may be needed according to availability and cost.

In some countries, CSF studies are the first to be performed. An example of how we work with our patients (not a recommendation) is shown in Figure 2, based on different scenarios according to CSF findings. To maximize confidence, we prefer using the A^+^T^+^ profile for confirming AD presence [153]. In the case of inconclusive results, we use the τ_P-181_/Aβ_42_ [171] or (if affordable due to high cost) a PET scan for Aβ_42_. By doing so, we try to combine the various criteria and guidelines.

It is noteworthy to highlight that, accordingly to the AA revised guidelines, hybrid ratios encompass CSF τ_P-181_/Aβ_42_, CSF τ_T_/Aβ_42_, CSF Aβ_42_/Aβ_40_, and plasma %τ_P-217_, without discrimination, even though the last two ratios represent distinct protein deposits, tau, and amyloid beta protein, respectively. Conversely, the first ones demonstrate the change in tau protein in relation to amyloid beta, assuming that τ_P-181_/Aβ_42_ is more specific for AD, instead of total tau, which is a general indicator of neuronal damage. The nomenclature is of great importance, considering it may help both the comprehension of ratio implications and their accurate utilization.

An AD diagnosis based only on biomarkers may not suffice for treatment eligibility. Some patients may have mixed pathologies, and AD may not be the main pathology responsible for the clinical picture. This may be true in cases with DLB [154] or significant cerebrovascular disease [159], and evidence or suspicion of such co-pathologies is considered a contraindication to anti-amyloid treatment [14].

The clinical phenotype of AD may also be important. For the common AD phenotypes (typical amnestic, logopenic-type primary progressive aphasia, and posterior cortical atrophy) [42], there are no concerns [16,173].

However, for the uncommon AD phenotypes (frontal variant, non-logopenic primary progressive aphasia, and corticobasal syndrome), AD may not be the only pathology, and, since the presence of other co-pathologies cannot be excluded, anti-amyloid treatments may not be indicated [16].

From this perspective, it is questionable whether blood-based biomarkers could be a standalone tool for AD diagnosis and evaluation of anti-amyloid treatment eligibility, despite the adequate diagnostic accuracy of some of them. We need further investigation into the plasma biomarker changes in the aforementioned mixed and atypical pathologies, as well as in other pathologies that can affect the central nervous system, including secondary causes of dementia. Moreover, a recent approach to their assessment, also classified according to the performed immunoassays [174], enlightens a new era with realistic expectations and skepticism.

There are some limitations in this review. We did not perform a systematic or umbrella review with or without meta-analyses. But this was not our aim. We preferred a simple narrative review to show the current uncertainties in biomarker-based confirmation of AD, not for theoretical reasons but for implementing anti-amyloid treatments or for recruiting patients for clinical trials of new drugs. Furthermore, the data enclosed and analyzed primarily come from White populations, limiting the potential of generalizability in different ancestries.

## 9. Future Directions

Currently, there are many criteria and guidelines for AD diagnosis [33,34,41,42,43,44,45] and for eligibility for anti-amyloid treatments [13,14,15,16,17,18]. Among them, there are many common features, but also some differences. Every effort should be made to reach a consensus and achieve homogeneous criteria.

To increase diagnostic accuracy, alternative biomarker testing for synaptic loss, microglia activation, and neuroinflammation should become more widely available since, in the appropriate clinical setting, they have the potential to offer additional information and explain some discrepancies between core biomarkers [119,128].

To simplify the diagnostic procedure and potentially decrease the cost, plasma biomarkers, especially τ_P-217_ and the τ_P-217_/Aβ_42_, are promising and may enter everyday diagnostic algorithms in the near future [16]. However, the recently published AA Clinical Practice Guideline suggested caution since there may be significant variability in their diagnostic accuracy and moderate confidence [174]. The role of salivary, urinary, or ocular biomarkers in offering simple and accurate diagnosis remains to be established [137].

## Figures and Tables

**Table 1:** Biomarkers of various types of abnormal biochemistry/pathology, useful in the (differential) diagnosis of Alzheimer’s disease and other dementia-related disorders, based on the classifications presented in Refs. [41,43] ^1^.

A (Amyloid)	T (Tau)		N (Neurodegeneration)	V (Vascular)	S (α-syn)	I (Inflammation)
	T_1_	T_2_				
Core 1		Core 2				
CSF Aβ_42_	CSF τ_P-181_	CSF τ_P-205_	CSF τ_T_		CSF α-syn	CSF GFAP
CSF Aβ_42_/Aβ_40_	CSF τ_P-217_	CSF MDBR-τ_243_	CSF NfL			
	CSF τ_P-231_	Other tau forms				
PET Aβ		PET tau	PET FDG			
			SPECT HMPAO			
			MRI ASL			
			Atrophy (MRI, CT)	MRI, CT		
Plasma Aβ_42_	Plasma τ_P-217_	Plasma eMDBR-τ_243_	Plasma NfL			Plasma GFAP
Plasma Aβ_42_/Aβ_40_	Plasma τ_P-181_		Plasma τ_T_			
	Plasma τ_P-212_					
Hybrid ratios or combinations of different markers, including CSF τ_P-181_/Aβ_42_, CSF τ_T_/Aβ_42_, and plasma τ_P-217_/Aβ_42_, are also in use.						

CSF: cerebrospinal fluid; PET: positron emission tomography; Aβ: amyloid beta peptide with 42 (Aβ_42_) or 40 (Aβ_40_) amino acids; τ_P-x_: phospho-tau phosphorylated at residue x; eMDBR-τ_243_: (endogenous cleaved) tau microtubule-binding region containing residue 243; τ_T_: total tau protein; NfL: neurofilament light protein; FDG: fluoro-deoxy-glucose; SPECT HMPAO: Single-Photon Emission Computed Tomography with ^99m^Tc-Hexamethylpropylen-amine Oxime; MRI: Magnetic Resonance Imaging; MRI ASL: MRI with arterial spin labeling; CT: Computerized Tomography; α-syn: alpha synuclein; GFAP: Glial Fibrillary Acidic Protein. ^1^ Other biomarkers may include CSF TDP-43 for transactive-response DNA-binding protein-43 (co)pathology and PET for α-syn or TDP-43.

## Data Availability

No new data were created or analyzed in this study. Data sharing is not applicable to this review article.
